# Preoperative dexamethasone for acute post-thoracotomy analgesia: a randomized, double-blind, placebo-controlled study

**DOI:** 10.1186/s12871-018-0599-0

**Published:** 2018-09-27

**Authors:** Kyoung-Woon Joung, Hyeong Ryul Kim, Wook-Jong Kim, Ye Ji Lim, Jae Won Kim, Eun-Ho Lee, In-Cheol Choi

**Affiliations:** 1 Department of Anesthesiology and Pain Medicine, Laboratory for Perioperative Outcomes Analysis and Research, Asan Medical Center, University of Ulsan College of Medicine, 88 Olympic-ro 43-gil, Songpa-gu, Seoul, 05505 Korea; 2 Department of Thoracic and Cardiovascular Surgery, Asan Medical Center, University of Ulsan College of Medicine, Seoul, Korea; 3 Department of Anesthesiology and Pain Medicine, Asan Medical Center, University of Ulsan College of Medicine, Seoul, Korea

**Keywords:** Analgesia, Dexamethasone, Epidural, Thoracotomy

## Abstract

**Background:**

The analgesic effects of dexamethasone have been reported previously, and the present study determined the effects of preoperative dexamethasone on postoperative pain in patients who received thoracotomy.

**Methods:**

Forty patients participated in this randomized, double-blind study. All patients received either dexamethasone via a 0.1 mg/kg intravenous bolus before anesthetic induction or an equal volume of saline. Postoperative analgesia was provided to both groups via epidural patient-controlled analgesia (PCA), which consisted of 250 μg of sufentanil in 250 mL of ropivacaine (0.18%) for 72 h. The primary outcome was the cumulative consumption of epidural PCA at postoperative 24 and 72 h. The secondary outcomes were the pain intensity scores during resting and coughing at postoperative 24 and 72 h, quality of recovery, total amount of rescue analgesics required, and length of hospital stay.

**Results:**

No significant differences was observed in the consumption of epidural PCA between the control and dexamethasone infusion groups at 24 h (63.6 [55.9–72.7] vs. 68.5 [60.2–89.0] ml, *P* = 0.281) and 72 h (199.4 [172.4–225.1] vs. 194.7 [169.1–252.2] ml, *P = 0*.890). Moreover, there was no significant difference in the pain intensity scores during resting and coughing at postoperative 24 and 72 h, quality of recovery, total amount of rescue analgesics required, and length of hospital stay.

**Conclusion:**

A single intravenous administration of dexamethasone during the preoperative period does not reduce opioid consumption and post-thoracotomy pain.

**Trial registration:**

The study was registered at http://cris.nih.go.kr (KCT0000359) and was conducted from December 2011 to October 2012.

## Background

Many patients experience intense pain following thoracotomy. Although thoracic epidural analgesia (TEA) is believed to be the most effective single technique for controlling post-thoracotomy pain [1], it often fails to treat post-thoracotomy pain. Thus, anesthesiologists require some adjuvant treatments for thoracotomy pain relief.

Recent studies have suggested that glucocorticoids are beneficial as postoperative analgesic agents because of their anti-inflammatory property in various surgeries [2–6]. Moreover, a recent meta-analysis suggested that dexamethasone is an effective adjunct to multimodal strategies for reducing postoperative pain and opioid consumption [7]. However, the efficacy of dexamethasone in controlling post-thoracotomy pain remains uncertain.

Our hypothesis is that the preoperative administration of dexamethasone may be an effective adjuvant treatment for post-thoracotomy pain like other surgeries. Thus, in this randomized, controlled double-blind study, we investigated whether the preoperative administration of this drug reduces post-thoracotomy pain during the recovery period.

## Methods

### Study population

After obtaining approval from our institutional review board (2011–0812) and written informed consent from each patient, 40 patients with American Society of Anesthesiologists physical status I–II for more than 20 years, who were scheduled for thoracotomy under general anesthesia, were enrolled in this prospective study. Exclusion criteria included an emergent infection for < 1 month before surgery; contraindications for dexamethasone or opioid use; severe cardiac, renal, or hepatic diseases; psychiatric disorder; uncontrolled diabetes mellitus; and the preoperative use of analgesics or steroids. The study was registered at http://cris.nih.go.kr (KCT0000359) and conducted between December 2011 and October 2012.

Patients were randomly allocated to two groups using computer-generated codes that were maintained in sequentially numbered opaque envelopes. On the morning of the surgery and before the induction of anesthesia, the envelopes were opened by a blinded nurse or anesthesiologist who subsequently prepared either dexamethasone or saline in coded 5-mL syringes. All other anesthesiologists involved in patient management or data collection were blinded to the group assignment. Just before the induction of anesthesia, each patient was administered an intravenous (IV) bolus injection of 0.1 mg/kg dexamethasone or an equal volume of saline.

### Anesthetic management and surgical technique

Before the anesthetic induction, all patients underwent thoracic epidural catheterization at the T6–7 intervertebral space using the loss-of-resistance technique. General anesthesia was induced using a bolus IV injection of 0.2 mg/kg etomidate and the continuous infusion of propofol and remifentanil using a target-controlled infusion pump. Furthermore, a bolus IV injection of 0.8 mg/kg rocuronium was administered to perform orotracheal intubation. After the intubation, patients were ventilated to normocapnia using 50% oxygen and air. The effect-site concentration of propofol was adjusted to maintain the bispectral index value between 40 and 60 (using 0.1–0.2 μg/mL steps), and remifentanil was administered to maintain the heart rate within 15% of the pre-induction value and systolic arterial blood pressure within 20% of the baseline value (using 0.5 ng/mL steps). If the mean arterial blood pressure and cardiac index showed a more than 20% decrease from the preoperative values, vasoconstrictors or inotropics, such as phenylephrine, dopamine, or norepinephrine, were administered. All patients underwent standard posterolateral thoracotomy by the same surgical team. The serratus anterior muscle was preserved, and the sixth rib was removed. During thoracotomy, all patients received one-lung ventilation using a double-lumen endotracheal tube. Postoperative analgesia was administered to both groups via epidural patient-controlled analgesia (PCA), consisting of 250 μg of sufentanil in 250 mL of ropivacaine (0.18%) administered as a 2-mL bolus dose with a 15-min lockout period; a maximum of 10 mL was administered over 1 h, and 2 mL of background infusion was maintained for 72 h postoperatively. Epidural PCA was started during the closure of the muscle layer. The administration of all drugs was stopped 5 min before the end of surgery, and extubation was performed in case of a response to verbal commands, spontaneous respiratory rate > 12 breaths/minutes, and end-tidal carbon dioxide partial pressure < 45 mmHg. Patients were admitted to the intensive care unit within 5 min of tracheal extubation. Both groups received rescue analgesics, if needed, during the postoperative period, and the total amount of rescue medication was recorded.

### Outcome evaluation

The primary outcome was the cumulative consumption of epidural PCA at postoperative 24 and 72 h. We believe that the cumulative consumption of epidural PCA is more objective than the pain score in assessing postoperative pain; therefore, we determined epidural PCA consumption as the primary outcome. The secondary outcomes included the pain intensity scores during resting and coughing, incidence of shoulder pain, total amount of rescue analgesics administered, and incidence of nausea and vomiting at postoperative 24 and 72 h, as well as the quality of recovery, duration of intensive care unit (ICU) stay and length of hospital stay. Moreover, we determined the incidence of postoperative complications, such as vocal cord palsy, wound infection, subcutaneous emphysema, pneumothorax, chylothorax, new onset of atrial fibrillation, and in-hospital death. Pain was assessed using the numeric rating scale, by asking patients to rate their pain on a scale of 0 (no pain) to 10 (disabling pain). The quality of postoperative recovery was assessed using the global Quality of Recovery-40 (QoR-40) questionnaire at postoperative 24 and 72 h. This questionnaire consisted of 40 questions that examined 5 domains of patient recovery, namely physical comfort, physical independence, psychological support, emotional state, and pain. The global QoR-40 scores range from 40 to 200, representing very poor to outstanding recovery. Poor recovery is defined as < 1 standard deviation below the group mean in ≥2 dimensions or a global QoR-40 score of < 1 standard deviation of the group mean. The postoperative consumption of rescue analgesics was converted to the equianalgesic dose of IV morphine [8].

### Statistical analysis

Based on 5 preliminary patients, we identified that the epidural PCA requirement during the first 24 h of surgery was 68.7 ± 20.2 ml. With a sample size of 17 patients in each group, a total of 34 patients were calculated to obtain a statistical power of 80% at a significance level of 0.05 (2-tailed) to reduce 30% of epidural PCA requirement. The Initiative on Methods, Measurement, and Pain Assessment in Clinical Trials (IMMPACT) II consensus recommended that a 30% reduction in pain from baseline can be considered significant in chronic pain clinical trial [9]. Thus, reduction by 30–35% in pain intensity was rated as minimal clinically important difference in postoperative pain [10, 11]. In these contexts, we decided that 30% was the expected reduction rate. To allow for 15% drop-out during the study period, we recruited a total of 40 patients. Continuous variables such as epidural PCA consumption at 24 and 72 h, pain intensity score at resting and coughing, rescue drug dose, ICU stay and hospital stay were presented as the mean ± standard deviation or medians with the range, and were analyzed using the paired *t* test or Mann–Whitney rank-sum U test. Categorical variables such as the incidence of shoulder pain, nausea, vomiting, vocal cord palsy, wound infection, respiratory complication, subcutaneous emphysema, pneumonia, chylothorax, in-hospital death and poor recovery profile at POD 1 and 3, and new onset of atrial fibrillation were presented as frequencies and percentages and assessed using the Pearson’s χ2 or Fisher’s exact test. Repeatedly measured data, such as QoR-40 and perioperative hemodynamics, were analyzed using 2-way analysis of variance. Statistical analyses were conducted using SPSS (Version 21.0; SPSS Inc., Chicago, IL, USA). In all comparisons, *P* < 0.05 was considered statistically significant.

## Results

**Fig. 1:**
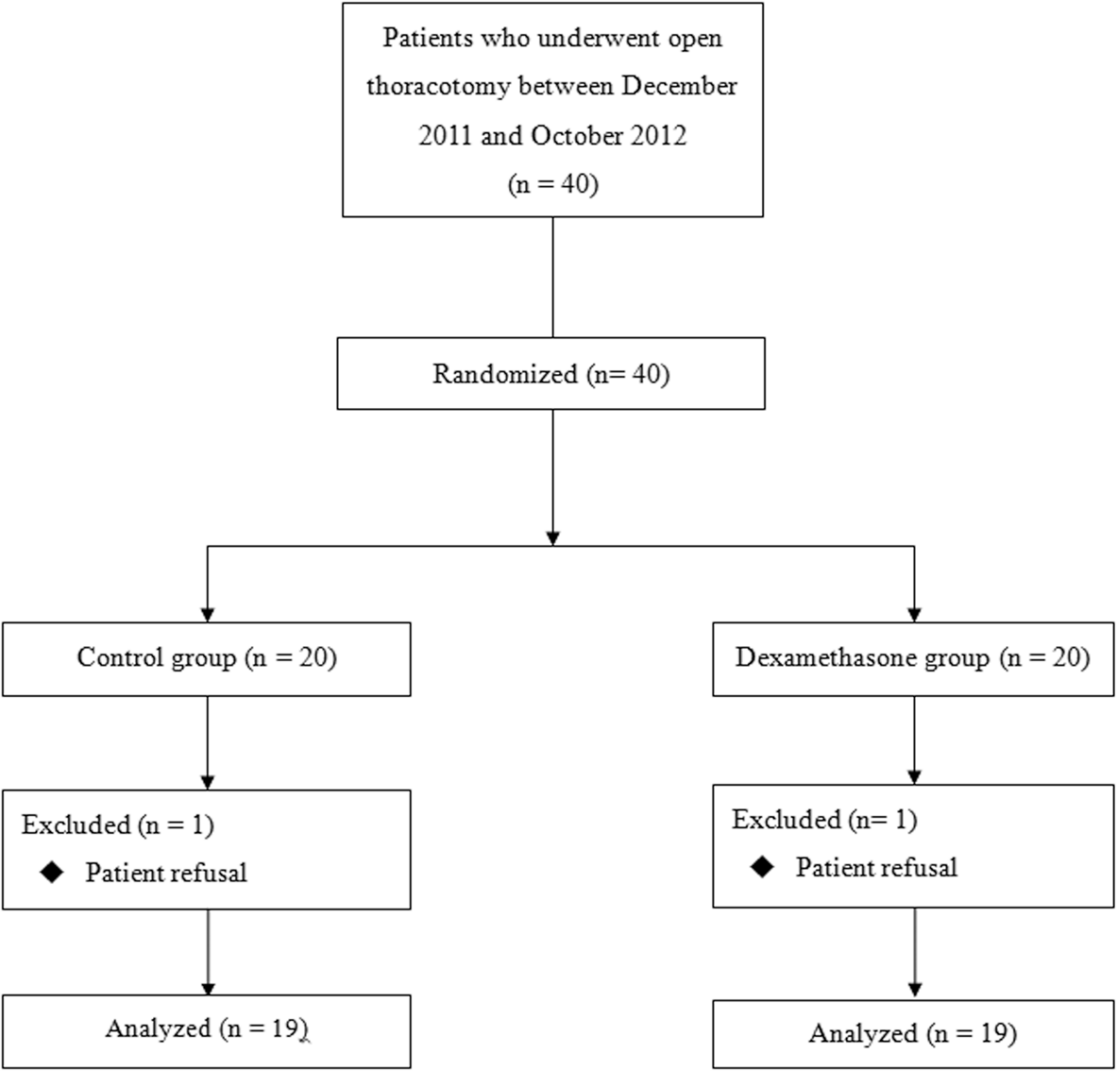
Study design according to the CONSORT statement

Between December 2011 and October 2012, 40 patients were enrolled in this prospective, randomized, double-blind study. All patients were randomly allocated to the control (group C) or dexamethasone (group D) groups. Among the enrolled patients, 2 were excluded because they did not want to participate (1 patient each in both groups). Therefore, 38 patients were included in the final analysis (Fig. 1).

In the final analysis, group C and D included 19 patients each. No significant differences were observed in the demographic data or perioperative characteristics of these groups (Table 1). The cumulative consumption of epidural PCA did not differ between groups C and D at 24 h (63.6 [55.9–72.7] vs. 68.5 [60.2–89.0] ml, *P* = 0.281) and 72 h (199.4 [172.4–225.1] vs. 194.7 [169.1–252.2] ml, *P* = 0.890). Other outcomes, such as the pain intensity score during resting and coughing, shoulder pain, postoperative consumption of rescue analgesics, and incidence of nausea and vomiting, were also not significantly different (Table 2). Moreover, the QoR-40 scores showed no significant differences between both global and individual categories (Table 3). No differences were observed in hospital stay, intensive care unit stay, or incidence of postoperative complications between the two groups (Table 4).

**Table 1:** Demographic and clinical characteristics of the subjects according to dexamethasone

	Control (*n* = 19)	Dexamethasone(*n* = 19)
Demographics		
Age (years)	65.60 ± 7.94	66.50 ± 8.44
Female sex	5 (26.3)	6 (31.6)
Body mass index (kg/m^2^)	24.20 ± 2.44	24.54 ± 2.48
ASA class		
I	5	2
II	14	17
Legion location		
Right upper lobe	6	6
Right middle lobe	2	1
Right lower lobe	5	6
Left upper lobe	1	2
Left lower lobe	5	4
Laboratory data		
Hematocrit (%)	39.23 ± 2.77	38.63 ± 2.92
Creatinine (mg/dL)	0.74 [0.64–0.84]	0.76 [0.67–0.98]
Albumin (g/dL)	3.80 [3.70–3.98]	3.85 [3.33–4.20]
C-reactive protein (mg/dL)	0.20 [0.10–1.78]	0.52 [0.11–1.48]
Glucose (mg/dL)	115 [107–142]	126 [106–156]
Sodium (mmol/L)	140 [137–142]	140 [137–142]
Potassium (mmol/L)	4.25 ± 0.21	4.29 ± 0.36
Pulmonary function test		
FVC (% predicted)	93.06 ± 16.07	85.19 ± 11.48
FEV_1_ (% predicted)	88.72 ± 20.31	81.52 ± 16.99
FEV_1_/FVC	69.05 ± 11.42	68.55 ± 10.60
Medical history		
Diabetes mellitus	3 (15.79)	5 (26.32)
Hypertension	5 (26.32)	6 (31.58)
Dyslipidemia	3 (15.79)	4 (21.05)
Smoker, current	5 (26.32)	4 (21.05)
Intraoperative data		
Anesthetic agent		
Propofol (mg)	1084.5 ± 301.4	1157.8 ± 390.4
Remifentanil (μg)	5530.4 ± 1879.1	6428.0 ± 3616.3
Anesthetic time (min)	237.5 [211.3–301.3]	257.5 [230.0–322.5]
Operation time (min)	165.0 [145.0–236.5]	192.5 [165.0–251.3]
Intraoperative infused volume		
Crystalloid (L)	1.0 [0.8–1.6]	1.1 [0.8–1.4]
Colloid (L)	0.6 [0–1.0]	0.6 [0.4–1.0]
Urine output (mL)	207.5 [132.5–377.5]	240.0 [112.5–802.5]
Use of vasopressor, n	12 (63.16)	11 (57.90)

Data are expressed as the number of patients (%), mean ± standard deviation, or median [first-third quartiles]

*ASA* American society of anesthesiology, *FVC* functional vital capacity, *FEV*_*1*_ forced expiratory volume in 1 s

**Table 2:** Primary and secondary outcomes according to dexamethasone administration

	POD 1			POD 3		
	Control (*n* = 19)	Dexamethasone (*n* = 19)	*p*	Control (*n* = 19)	Dexamethasone (*n* = 19)	*p*
Epidural PCA (mL)	63.6 [55.9–72.7]	68.5 [60.2–89.0]	0.281	199.4 [172.4–225.1]	194.7 [169.1–252.2]	0.890
NRS						
Rest	3.0 [1.4–4.0]	3.0 [1.0–4.5]	0.975	2 [1.5–3.0]	2 [1.0–2.5]	0.549
Cough	3.0 [3.0–8.0]	3.8 [3.0–5.8]	0.781	3.0 [3.0–6.0]	3.0 [2.0–5.0]	0.369
Shoulder pain	5 (26.32%)	2 (10.53%)	0.405	4 (21.05%)	3 (15.79%)	1.000
Rescue drug (mg)	10 [10–20]	10 [10–20]	0.453	2.5 [0–26]	2.5 [0–19]	0.862
Nausea	2 (10.53%)	2 (10.53%)	1.000	1 (5.26%)	1 (5.26%)	1.000
Vomiting	0 (0%)	1 (5.26%)	1.000	0 (0%)	0 (0%)	1.000

Data are expressed as the number of patients (%) or median [first-third quartiles]

*POD* post-operative day, *PCA* patient-controlled analgesia, *NRS* numerical rating scale

**Table 3:** Quality of Recovery-40 scores according to dexamethasone administration

	Preoperative	POD1	POD3	*p*
QoR_Global				
Control	187 [176–194]	174 [164–189]	189 [178–191]	0.625
Dexamethasone	187 [166–195]	182 [170–193]	190 [177–194]	
Physical comfort				
Control	56 [54–59]	53 [48–56]	57 [54–58]	0.857
Dexamethasone	56 [53–60]	55 [51–59]	57 [50–60]	
Physical independence				
Control	25 [25–25]	20 [15–22]	22 [19–25]	0.213
Dexamethasone	25 [23–25]	25 [18–25]	23 [21–25]	
Psychological support				
Control	33 [31–35]	35 [29–35]	35 [34–35]	0.625
Dexamethasone	33 [29–35]	35 [34–35]	35 [35–35]	
Emotional state				
Control	42 [41–43]	42 [38–45]	43 [42–45]	0.967
Dexamethasone	41 [36–44]	42 [39–45]	44 [40–45]	
Pain				
Control	35 [32–35]	32 [27–34]	32 [29–35]	0.940
Dexamethasone	35 [32–35]	31 [28–33]	33 [31–35]	

Data are expressed as the median [first-third quartiles]

*QoR* Quality of Recovery, *POD* post-operative day

**Table 4:** Post-operative outcomes according to dexamethasone administration

	Control (*n* = 19)	Dexamethasone (*n* = 19)	*p*
ICU stay (hours)	21.5 [17.3–24.0]	22.5 [20.0–23.0]	0.643
Hospital stay (days)	6 [5–8]	6 [5–8]	0.967
Shoulder pain	7 (36.84%)	4 (21.05%)	0.474
Vocal cord palsy	3 (15.79%)	0 (0)	0.230
Wound infection	1 (5.26%)	0 (0)	1.000
Respiratory complication	5 (26.32%)	7 (36.84%)	0.727
Subcutaneous emphysema	1 (5.26%)	2 (10.53%)	1.000
Pneumonia	1 (5.26%)	4 (21.05%)	0.340
Chylothorax	2 (10.53%)	2 (10.53%)	1.000
In-hospital death	0 (0%)	0 (0%)	1.000
Rescue analgesic drug, total (mg)	25 [10–39]	18 [10–35]	0.549
Poor QoR day1	15 (78.9%)	10 (52.6%)	0.171
Poor QoR day3	13 (68.4%)	10 (52.6%)	0.507
Postoperative atrial fibrillation	4 (21.05%)	1 (5.26%)	0.340

Data are expressed as the number of patients (%) or median [first-third quartiles]

*ICU* intensive care unit, *QoR* quality of recovery

## Discussion

The present study shows that a preemptive single administration of dexamethasone (0.1 mg/kg) does not reduce the cumulative epidural PCA consumption during postoperative period.

To treat post-thoracotomy pain, we believe that the multimodal approach is important because pain after thoracotomy is caused by a multifactorial pathophysiology. The mechanisms underlying post-thoracotomy pain include nociceptive somatic and visceral mechanisms, neuropathic mechanisms, and referred pain. Somatic tissue injuries are caused by factors such as skin incision; rib retraction; muscle splitting; and injury of parietal pleura afferent through intercostal nerves and ipsilateral dorsal horn of the spinal cord and are transmitted to the limbic system and somatosensory cortex of the contralateral anterolateral system of the spinal cord. Injury to visceral tissues, such as bronchi, visceral pleura, and pericardium, are transmitted via phrenic and vagus nerves. These nociceptive stimuli increase inflammatory mediators, which is directly activate and enhance the nociceptor activity. This mechanism is called primary sensitization and causes acute pain after thoracotomy [12, 13]. The most common TEA regimen is a combination of local anesthetics and opioids. Local anesthetics in TEA enhance the bioavailability of opioids in the cerebrospinal fluid, increase binding to the mu receptor, and reduce the release of inflammatory mediators in the spinal cord. However, because the main mechanism of TEA is to block the central transmission of nociceptive stimuli, it does not block the peripheral nociception itself. Moreover, TEA does not reduce referred pain. Thus, the addition of local anesthetics or combination of anti-inflammatory drugs is considered to be a good multimodal approach to treat thoracotomy pain because it blocks peripheral nociception [12, 14, 15].

In our knowledge, to date, no study has reported the effect of the combination of epidural analgesia and IV steroids on post-thoracotomy pain. The pain relief properties of steroids are well established in metastatic bone, neuropathic, and visceral pain. Glucocorticoids confer anti-inflammatory effects by inhibiting prostaglandin synthesis and reduce tissue edema by modulating vascular permeability. Moreover, steroids reduce the spontaneous discharge of injured nerves [16, 17]. Dexamethasone is the most widely used glucocorticoid for the management of pain because it has less mineralocorticoid effects, is relatively more potent, and has a longer half-life than do other corticosteroids [18]. Some studies have suggested that the single use of dexamethasone effectively alleviates postoperative pain as an adjuvant treatment option. Feroci et al. [4] reported that dexamethasone reduces the incidence of postoperative nausea and vomiting (PONV) and post-thyroidectomy pain. Fukami et al. [5] also reported that preoperative dexamethasone reduces the incidence of PONV, fatigue, and pain after laparoscopic cholecystectomy. Similar results have been reported in other surgeries, such as orofacial, urethral, and orthopedic surgeries [2, 19–22].

However, dexamethasone showed no analgesic effects in the present study. These results may be explained by the doses of dexamethasone. We used a relatively small dose of the drug (0.1 mg/kg) because corticosteroids may have various side effects, such as weight gain, muscle weakness, insomnia, and infection [18]. A recent meta-analysis reported that low-dose dexamethasone (≤0.1 mg/kg) does not reduce postoperative pain, although perioperative high-dose dexamethasone (> 0.1 mg/kg) confers this effect [23]. Moreover, the incidences of referred pain and PONV in this study were not different in the two groups. As mentioned earlier, TEA itself does not reduce referred pain, if preemptive dexamethasone is effective, postoperative referred pain should be reduce. With respect to PONV prevention, there is no need for further mentioning the effects of dexamethasone. Thus, we believe that the effect of a single, relatively small dose of dexamethasone is insufficient in treating thoracotomy pain. Another reason for the results may be explained that the “single” administration of dexamethasone in the preoperative period. Senard et al. [24] reported that the combination of TEA and parenteral cyclooxygenase (COX)-2 inhibitor (NSAID) reduces post-thoracotomy pain. We believe that dexamethasone and NSAIDs have similar mechanisms for treating post-thoracotomy pain by inhibiting COX. However, the main results reported by Senard and those of the present study are different. In Senard’s study, patients received parenteral COX-2 from the evening before surgery until postoperative 48 h. Craig et al. [25] reported that the inflammatory response after a thoracotomy incision peaked at approximately 48 h after surgery. Therefore, a preemptive single administration of dexamethasone may be insufficient in controlling post-thoracotomy pain. Lastly, in the present study, the effect of dexamethasone might be masked by the highly potent TEA regimen. This theory may explain the results of QoR-40. The QoR-40 scores did not differ between the two groups, and there was no difference between preoperative and postoperative periods either. This suggests that pain management for thoracotomy in the present study may be enough for the TEA itself.

The present study has some limitations. First, we did not perform a dose-dependent analysis and did not examine the effects of multiple doses. Second, we did not measure the pain intensity score during the immediate postoperative period. Because patients are unstable immediately after thoracotomy, the measurement of the pain intensity score might not be feasible. Furthermore, we administered epidural PCA during the closure of the muscle layer because the pain-reducing effects of epidural PCA are so effective that the combination effects of dexamethasone are often masked. Third, we did not examine chronic, neuropathic pain. Nociceptive stimuli occur continuously during the perioperative period; it leads to the hyperexcitability of the dorsal horn and may develop progress chronic, neuropathic pain. Dexamethasone reduces the spontaneous discharge of injured nerves. Our finding reflects that dexamethasone may be more effective against chronic, neuropathic pain than against acute pain. Finally, we only included 40 patients and acknowledged that additional, larger, dose-dependent, and time-dependent studies are required to elucidate the effects of dexamethasone on post-thoracotomy pain. To overcome these limitations, we think that dose-dependent, long-term outcome study should be needed to evaluate the effects of dexamethasone on post-thoracotomy pain.

## Conclusion

The single administration of dexamethasone during the preoperative period does not reduce post-thoracotomy pain during the recovery period.

## Abbreviations

COX-2: Cyclo-oxygenase 2
ICU: Intensive care unit
PCA: Patient-controlled analgesia
QoR: Quality of recovery
